# Intestinal organoid/enteroid-based models for *Cryptosporidium*

**DOI:** 10.1016/j.mib.2020.10.002

**Published:** 2020-12

**Authors:** Seema Bhalchandra, Hymlaire Lamisere, Honorine Ward

**Affiliations:** 1 Tufts Medical Center, Tufts University School of Medicine, Boston, MA, USA; 2 Tufts University Graduate School of Biomedical Sciences, Boston, MA, USA

## Abstract

*Cryptosporidium* is a leading cause of diarrhea and death in young children and untreated AIDS patients in resource-poor settings, and of waterborne outbreaks of disease in developed countries. However, there is no consistently effective treatment for vulnerable populations. Progress towards development of therapeutics for cryptosporidiosis has been hampered by lack of optimal culture systems to study it. New advances in organoid/enteroid technology have contributed to improved platforms to culture and propagate *Cryptosporidium*. Here we discuss recent breakthroughs in the field and highlight different models for functional *ex vivo* organoid or enteroidderived culture systems. These systems will lead to a better understanding of the mechanisms of host–parasite interactions *in vivo*.

**Current Opinion in Microbiology** 2020, **58**:124–129

This review comes from a themed issue on **Host–microbe interactions: parasites**

Edited by **Honorine Ward** and **Kami Kim**

For a complete overview see the Issue and the Editorial

Available online 25th October 2020

**https://doi.org/10.1016/j.mib.2020.10.002**

1369-5274/© 2020 The Authors. Published by Elsevier Ltd. This is an open access article under the CC BY license (http://creativecommons.org/licenses/by/4.0/).

## Introduction

*Cryptosporidium* is a highly infectious, intestinal protozoan parasite of medical and veterinary importance [1, 2, 3]. This parasite causes life-threatening diarrheal disease (cryptosporidiosis) in young children living in developing countries and in immunocompromised individuals, particularly those with untreated HIV/AIDS [3,4]. Reported cases in industrialized countries are also rising due to the major role that *Cryptosporidium* plays as a cause of waterborne outbreaks of disease [5]. Despite the global burden of cryptosporidiosis, treatment options are limited. Nitazoxanide, the only US Federal Drug Administration-approved drug is not effective in immunodeficient patients and not approved in infants under 1 year of age [6].

## Limitations in culture and propagation of Cryptosporidium *in vitro*

Progress in understanding the pathophysiology of cryptosporidiosis and in developing new interventions against *Cryptosporidium* has been hampered by a lack of optimal *in vitro* culture systems to recapitulate *in vivo* infection [7]. Genetic manipulation of *Cryptosporidium parvum* has recently been established using *CRiSPR/Cas9*-mediated gene targeting [8,9] and chemotherapeutic targets and vaccine candidates have been identified. However, transgenic parasites need to be selected and passaged in immunodeficient mice because they cannot be continuously propagated in culture *in vitro*.

Much of our present knowledge has resulted from studies with animal models or cultured immortalized cell lines that do not support completion of the parasite life cycle [10,11]. Compartmentalized three dimensional (3D) intestinal models using colon cancer-derived transformed HCT-8 cells provide an alternative culture system for continuous propagation [12,13]. However, these models require specialized equipment and are not amenable to basic imaging techniques or drug screening assays. Although the HCT-8 cell line supports a high rate of *C. parvum* infection [14], a block in gamete fusion can prevent the development of new oocysts thereby arresting parasite growth [15]. Self-regenerating primary human IEC cultivated from small intestinal crypts have been reported to extend the *in vitro* infection period of *C. parvum* for up to five days [16] and improved infection kinetics of *C. parvum* in a commercially available non-carcinoma, human IEC line has also been demonstrated [17]. However, these models do not support long term cultivation of the parasite.

Recently, we reported the successful culture of *C. parvum* in a novel 3D bioengineered silk scaffolding system utilizing colon cancer-derived, transformed Caco-2 and HT29-MTX cells [18]. This system supported continuous culture for 17 days and luminal contents could re-establish infection in fresh scaffolds to produce new oocysts *in vitro*. However, gene expression profiles of transformed IEC lines vary, and data generated from their use often does not correlate with physiological responses *in vivo*. The genotype of subcloned cell lines, especially Caco-2 cells, changes with increasing passage numbers or with differing culture conditions [19]. A 3D culture model developed from colonic explants of adult SCID mice [20] supported *C. parvum* infection for up to 27 days but is limited by the short lifespan of tissue explants. Given these shortcomings, the field has sought more physiologically relevant model systems to study *Cryptosporidium* infections [7,21,22]. Recent advances in stem cell biology have led to the development of organoids as a powerful, alternative model of intestinal physiology [23,24^••^]. In this review we focus on recent 2D and 3D organoid models that have been successfully used to culture and propagate *C. parvum* and interrogate aspects of the host–parasite interactions.

## Intestinal organoids/enteroids for *ex vivo* culture

Development of organoid technology to generate human ‘mini-intestines’ offers a unique potential for overcoming the inherent limitations of previous models. Seminal work in the past decade [25,26] has enabled long-term *ex vivo* culture of intestinal epithelial cells (IECs) from LGR5+ intestinal stem cells (ISC) derived from murine and human surgical specimens. ISCs formed self-organizing, self-renewing organ like structures (organoids) that can be propagated indefinitely while retaining tissue specific transcriptional and epigenetic profiles [27,28]. Organoids are maintained in culture by the addition of exogenous Wnt protein and can differentiate into absorptive and secretory cell lineages upon removal of Wnt and other factors that prevent cell differentiation [29]. Current nomenclature guidelines from the Intestinal Stem Cell Consortium [30] define ISC-derived epithelial only structures from tissues as enteroids in order to distinguish them from organoids that are derived from induced pluripotent stem cells. However, some investigators still refer to ISC-derived structures as organoids. In this review, we have used organoid/enteroid terminology as employed in the original paper.

Intestinal organoids or enteroids can be cultured in different formats, depending on the nature of the studies they are used for. When embedded in or cultured on top of extracellular matrix materials such as Matrigel, cells can self-assemble into 3D enclosed structures [31]. However, this structure is topologically constrained, rendering the lumen inaccessible, although the recent development of ‘apical-out’ cultures [32] has addressed this limitation. Given their versatility, ISC-based intestinal organoids or enteroids in various 2 or 3 D formats have been used successfully for *ex vivo* culture and/or investigation of host–pathogen interactions of a number of enteric pathogens including viruses, bacteria and parasites [24^••^,^33^, ^34^, ^35^, ^36^].

## Intestinal organoids/enteroids for *Cryptosporidium* culture and propagation

### Human intestinal organoid/enteroid models

**Figure 1:**
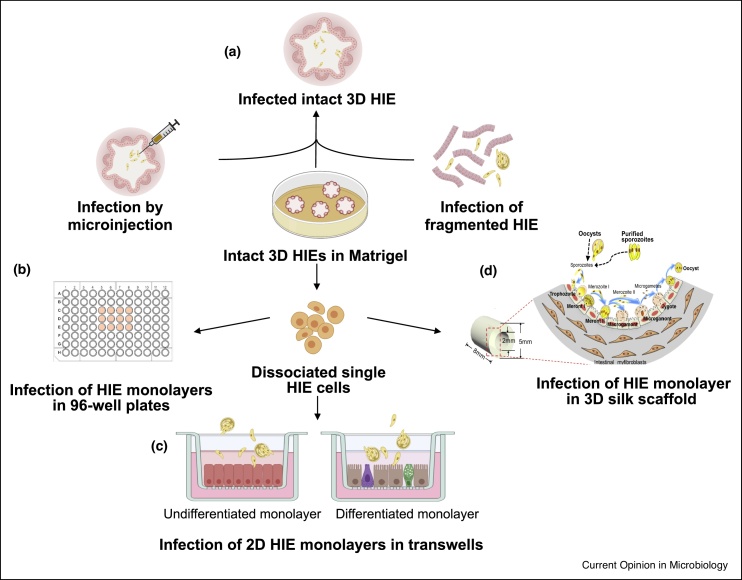
HIE models of *C. parvum* infection: **(a)** Intact 3D HIEs grown in Matrigel, are fragmented, incubated with *C. parvum* and re-plated in fresh Matrigel. Alternatively, HIE are dissociated into single cells and grown as 2D monolayers in **(b)** micro-titer plates or **(c)** transwell filters and infected apically with *C. parvum* oocysts or filtered sporozoites. **(d)** 3D silk scaffolds with luminal HIE monolayers are infected by introducing *C. parvum* oocysts or purified sporozoites into the lumen. Figure (d) modified and reproduced from Ref. [19] with permission from the American Society for Microbiology. Figure created in part with BioRender.com.

We recently established 2D and 3D models of cryptosporidiosis using human intestinal enteroids (HIE) derived from intestinal tissue biopsies [37^•^] (Figure 1). Intact, 3D HIE (undifferentiated or differentiated) are fragmented by vigorous pipetting in the presence of pancreatin followed by addition of *C. parvum* oocysts or sporozoites to enable parasite invasion through the apical epithelial surface (Figure 1a). Infection of intact 3D HIE can also be achieved by microinjection [38^••^]. To generate cultures with an accessible luminal surface for infection, intact HIE are dissociated into single cells following trypsinization and monolayers are established on collagen coated 96-well plates or 384-well plates (Figure 1b). This planar monolayer is amenable to high-throughput screening of small molecules or other drugs using glass-bottomed microtiter plates [39]. On transwell membranes, dissociated cells form a highly polarized 2D monolayer with defined apical and basolateral surfaces (Figure 1c). This system permits analysis of trans epithelial electrical resistance [40], imaging studies as well as access to the basolateral compartment. We also adapted the previously described bioengineered 3D human intestinal tissue model utilizing a silk protein scaffold [18,41] for *C. parvum* infection (Figure 1d). The geometrically engineered hollow lumen recapitulates the structure of the human small intestine and provides an improved *ex vivo* model to support *C. parvum* infection. Dissociated cells seeded circumferentially on the surface of the lumen are nourished by intestinal myofibroblasts dispersed in the porous scaffold bulk to establish a polarized monolayer and infection is achieved by introducing *C. parvum* oocysts or purified sporozoites into the lumen. This model can successfully support *C. parvum* infection (unpublished data). These HIE-based models offer tools for better investigating the interaction between *C. parvum* and the host intestinal epithelium during the course of infection.

In a landmark study, Heo *et al.* [38^••^] reported the entire life cycle progression of *C. parvum* in human organoids. The authors used small intestinal and lung organoids from healthy human donors to model the infection. Differentiated intact 3D intestinal organoids microinjected with sporozoites supported the full replicative cycle resulting in newly generated oocysts that were infectious to neonatal mice. Conversely, in medium containing growth factors, undifferentiated organoids were less susceptible to *C. parvum* highlighting a potential cell-type specific response to infection. RNA sequencing and transcriptome analysis revealed dynamic regulation of the parasite’s transcriptome during invasion. An upregulation of genes associated with type I interferon (IFN) pathway in response to parasite infection was observed in both lung and intestinal organoids. Produced early during an infection, type I IFNs have been described as a mediator of epithelial defense against viral pathogens. *C. parvum* has also been shown to induce type III IFNs in transformed IEC [42] but their role in infection needs further investigation. In a novel finding, organoids derived from bronchial airways could support *C. parvum* infection, thereby providing a valuable tool for recapitulating lung epithelial infections of the poorly studied lung respiratory route of human disease transmission [3]. Transmission electron microscopy demonstrated that the parasite could infect both absorptive enterocytes and secretory cell types in lung organoids. However, this study was limited in investigating invasion of the parasite in real time and cannot be utilized in large-scale drug screenings. The parasites persisted within organoids for 28 days with serial passaging, but the yield of oocysts was found to decline significantly over time thereby limiting the possibility for long-term continuous propagation in organoids. Confined in a 3D matrix that lacks perfusion, cystic organoids cease to proliferate upon reaching a certain size and develop a necrotic core; hence cannot be used to model long-term infection.

### Murine intestinal organoid/enteroid models

Development of murine enteroids that recapitulate integral aspects of the host environment have provided useful insights into the impact of *C. parvum* infection on the intestinal epithelium. Zhang *et al.* [43] introduced an *ex vivo* model of cryptosporidiosis utilizing 3D enteroids derived from neonatal and adult mice. *C. parvum* infection for 48 hour led to decreased bud formation (a marker of proliferation) and inhibition of enteroid propagation compared to uninfected controls. Decreased expression levels of intestinal stem cell markers Lgr5 and Sox9 observed by real time-PCR and immunohistochemistry suggested that parasite infection could involve inhibition of ISC function through attenuation of the Wnt/B-catenin signaling resulting in apoptotic cell death and senescence. However, further investigation is required to support this hypothesis.

Enteric infections can affect the ISC niche and organoids/enteroids provide a reliable system to investigate factors contributing to infection-induced diarrhea. Our group and others documented disruption of the intestinal barrier integrity in cryptosporidiosis in children [44] which has been confirmed *in vitro* in murine enteroids [45]. A recent study [45] examined the effects of *C. parvum* infection on paracellular permeability and expression of key epithelial tight junction and adherens junction proteins in murine organoid-derived monolayers cultured on transwells. The authors found downregulation of expression of occludin, claudins 3 and 4 and E-cadherin following *C. parvum* infection for 24 hours. More extensive studies in human enteroid models will be necessary to determine the mechanisms involved in disruption of intestinal integrity and epithelial repair in *C. parvum* infection.

Wilke *et al.* recently described a murine ileum stem cell-derived culture system in an experimental setup that favored development of an air–liquid interface (ALI) [46^••^,^47^]. Under these conditions, murine organoid monolayers supported by 3T3 fibroblast feeder cells formed differentiated monolayers on transwells. Enhanced host cell differentiation attributed to an upregulation of cell cycle and metabolic pathways supported complete life cycle development and long-term growth of *C. parvum.* A 100-fold increase in parasite load was observed, generating *de novo* oocysts that were infectious *in vitro* and in IFN-γ receptor deficient mice. Using a panel of stage-specific monoclonal antibodies [48^•^], all previously described asexual and asexual stages as well as a late stage macrogamonts containing wall forming bodies were identified. Additionally, the authors created transgenic parasite lines using *CRiSPR/Cas9* technology and performed a genetic cross *in vitro*, to produce viable, recombinant parasites, which is currently not possible with other apicomplexan parasites. In another recent report, the long-term murine ALI organoid model capable of supporting continuous parasite growth has proved promising in studying cidal activity and mechanism of action of small molecule inhibitors of *C. parvum* infection [49].

## Conclusion and future directions

Recent technological advances in successful cultivation and propagation of *C. parvum* in murine and human organoids/enteroids provide an exciting new avenue for understanding *Cryptosporidium* parasites and their biology. These platforms will further enable study of the molecular basis of the life cycle changes, host–parasite interactions and identification of crucial functional genes and their roles as possible therapeutic targets. However, much progress remains to be made.

*Ex vivo* organoid/enteroid models can be further refined to permit selection and propagation of transgenic parasites in culture which is currently not possible and relies on the use of immunodeficient mice [9]. Advances in propagation of *C. parvum* in *ex vivo* human organoid/enteroid cultures can be applied to the culture of the anthroponotic species *Cryptosporidium hominis*, the major species infecting humans [50] and which can only be propagated with difficulty in gnotobiotic piglets and calves [50].

Increasing the complexity of organoid/enteroid cultures by including components of the *in vivo* host tissue microenvironment such as immune, endothelial and neural cells as well as the gut microbiome [28] will further facilitate studies on pathogenesis, pre-clinical evaluation of vaccines, and therapeutics for cryptosporidiosis. Use of the ‘apical’ out system [32] may provide a more physiological model. These advanced, *ex vivo* culture models can also be used to study regenerative responses to intestinal damage [51] resulting from *Cryptosporidium* infection. Combining bioengineering technologies like microfluidics [52] to incorporate perfusion, flow and tunable oxygen levels will further mimic physiological conditions [53]. Further research based on new platforms such as the recently developed duodenum Intestine-Chip [54], may also contribute to improved therapeutics for cryptosporidiosis.

## Conflict of interest statement

Nothing declared.

## References and recommended reading

Papers of particular interest, published within the period of review, have been highlighted as:

• of special interest
•• of outstanding interest

## CRediT authorship contribution statement

**Seema Bhalchandra:** Conceptualization, Vizualization, Methodology, Writing - original draft, Writing - review & editing. **Hymlaire Lamisere:** Conceptualization, Writing - review & editing. **Honorine Ward:** Conceptualization, Project administration, Resources, Writing - review & editing.
